# Identification of a Hypoxia-Related Gene Signature for Predicting Systemic Metastasis in Prostate Cancer

**DOI:** 10.3389/fcell.2021.696364

**Published:** 2021-10-13

**Authors:** Haoran Xia, Jianlong Wang, Xiaoxiao Guo, Zhengtong Lv, Jingchao Liu, Qiuxia Yan, Ming Liu, Jianye Wang

**Affiliations:** ^1^Department of Urology, Beijing Hospital, National Center of Gerontology, Institute of Geriatric Medicine, Chinese Academy of Medical Sciences, Beijing, China; ^2^Graduate School of Peking Union Medical College, Chinese Academy of Medical Sciences, Beijing, China; ^3^Fifth School of Clinical Medicine, Peking University, Beijing, China

**Keywords:** hypoxia, prostate cancer, gene signature, metastasis, therapeutic resistance, immune infiltration

## Abstract

**Background:** Systemic metastasis is the main cause of death in patients with prostate cancer. It is necessary to establish a more accurate model to distinguish and predict patients with a high risk of metastasis to optimize individualized treatment.

**Methods:** In this study, it was determined that hypoxia could affect the metastasis-free survival of patients with prostate cancer, and a hypoxia-related gene signature composed of seven genes for predicting metastasis was established and verified in different cohorts. The study further evaluated the effects of *ALDOB* expression on the proliferation and invasion of the LNCaP and DU145 cell lines under hypoxia and finally constructed a nomogram containing specific clinical characteristics of prostate cancer combined with the hypoxia gene signature to quantify the metastasis risk of individual patients.

**Results:** The hypoxia-related gene signature was identified as an independent risk factor for metastasis-free survival in patients with prostate cancer. The expression of *ALDOB* increased under hypoxia and promoted the proliferation and invasion of LNCaP and DU145 cells. In addition, patients with a high risk score showed therapeutic resistance and immunosuppression. Compared with other parameters, the nomogram had the strongest predictive power and net clinical benefit.

**Conclusion:** The study established a hypoxia-related gene signature and a nomogram to distinguish and predict patients with a high risk of prostate cancer metastasis, which may help to optimize individualized treatment and explore possible therapeutic targets.

## Introduction

Prostate cancer is a global health threat. According to statistics published in 2020, among the 19.3 million new cancer cases, there were 1,414,259 cases of prostate cancer, accounting for 7.3% of the total cases and ranking third among 36 cancers (Sung et al., 2021). Of those diagnosed with prostate cancer, 3.8% face death, and systemic metastases remain the leading cause of death (Sung et al., 2021). In recent years, the diagnosis and treatment of prostate cancer have been continuously developed and improved. For example, prostate-specific antigen (PSA) is used for early screening of disease, and treatment is carried out according to clinical features, such as the Gleason score (GS) and tumor TNM stage. After treatment, the vast majority of prostate cancer patients will eventually enter the castration-resistant prostate cancer (CRPC) stage. However, there is insufficient evidence to show that these clinical features are sufficient to describe tumor invasion and metastasis (Prensner et al., 2012). Therefore, it is necessary to identify a more sensitive and reliable predictor to highlight patients with a high risk of metastasis for more appropriate and individualized systemic treatment.

In cancer tissue, the imbalance in the growth and necrosis of cells often leads to a significant increase in oxygen demand, creating a hypoxic microenvironment that results in inflammation, angiogenesis and other reactions to promote the development of cancer and eventually forms a vicious cycle (Semenza, 2012; Gilkes and Semenza, 2013; Gilkes et al., 2014). In the field of prostate cancer research, hypoxia gene expression patterns have been shown to be significantly different between primary and metastatic prostate cancer (Bharti et al., 2019). Hypoxia can not only affect the expression of cancer-related genes (Khandrika et al., 2009; Yamasaki et al., 2013; Bowler et al., 2018; Zheng and Bai, 2019) but also promote the proliferation, migration and invasion of prostate cancer by activating a variety of signaling pathways or promoting stem cell properties (Hugo et al., 2007; Ma et al., 2011; Yang et al., 2020). Using hypoxia-related genes as markers may help to distinguish patients with a high risk of metastasis.

In this study, hypoxia-related genes were screened according to published gene expression information to predict prostate cancer metastasis. Validation was carried out in different test sets, and then treatment response and immune infiltration were analyzed. Finally, by combining the hypoxia gene signature and clinical characteristics, a reliable model was established, which ensured a high predictive ability in the horizontal comparison of existing risk factors.

## Materials and Methods

### Download and Preparation of a Patient Dataset

A total of 1,325 different-stage prostate cancer patients with gene expression, clinical annotation and follow-up data from The Cancer Genome Atlas (TCGA) and Gene Expression Omnibus database (GEO) platforms were included in the study. Among the patients, 481 from the TCGA platform were used as the training set, and their fragments per kilobase per million mapped reads (FPKM)-normalized RNA-Seq data and follow-up information were downloaded to construct a hypoxia-related gene predictive model. The GSE116918 dataset, consisting of a cohort of localized/locally advanced prostate cancer patients commencing radical radiotherapy (with androgen deprivation therapy) and generated by the Almac Diagnostics Prostate Disease Specific Array (DSA) chip platform, was downloaded from GEO^1^, and the 248 patients in the dataset were used as the validation set for the model. In addition, 596 patients receiving radical retropubic prostatectomy (RRP) from the GSE10645 dataset (DASL Custom Prostate Panel by Gene Chip Platform^2^) were used as supplementary independent validation cohorts. For the gene expression data in all datasets, data normalization and log2 transformation were necessary.

### Screening of Candidate Hypoxia Genes and Model Construction

First, according to the Molecular Signatures Database (MsigDB)^3^ HALLMARKS cancer characteristic gene set, the scores for 50 pathological pathways in each sample of the training set were calculated using the “zscore” algorithm of the single sample gene set enrichment analysis (ssGSEA) (R package “GSVA”) (Lee et al., 2008). Then, a hypoxia-related protein-protein interaction (PPI) network was built by the String PPI website^4^. The number of connecting nodes for each gene was calculated, and the first 50 genes were selected as hypoxia core genes and intersected in the expression matrix of the TCGA and GEO datasets. After batch correction, the expression matrix of the core hypoxia genes in the TCGA and GEO was constructed. A univariate Cox proportional hazard regression model was applied by loading the R package “survival,” and 20 candidate genes were selected for inclusion in least absolute shrinkage and selection operator (LASSO) Cox regression (R package “glmnet”) and multivariate Cox stepwise regression models (R package “survival”) (Tibshirani, 1997). Then, the genes with a high correlation and duplicate information were removed to optimize the model, and finally, a hypoxia risk score was obtained by multiplying the results for each gene’s coefficient and expression in the matrix.

### Statistical and Bioinformatic Analyses

R software (version 4.0.3^5^) and GraphPad Prism 8 were used to perform data analysis and graphic visualization. According to the median hypoxia risk score, patients were divided into a high-risk group and a low-risk group. Using the R package “ConsensusClusterPlus” to perform non-negative matrix factorization (NMF) consensus clustering, the supplementary cohort was divided into different groups according to the characteristics of the remaining hypoxia genes. Heat maps, histograms, scatter charts and Sankey diagrams were used to show gene expression and prognosis in different groups (R packages “reshape2,” “ggplot2,” “scales,” “cowplot,” “ggalluvial,” and “sankeyNetwork”), and the differences between groups were analyzed by *t*-tests. Principal component analysis (PCA) and t-distributed stochastic neighbor embedding (tSNE) algorithms were used to reduce dimensionality to show the degree of differentiation between two groups (R package “Rtsne”) (Reich et al., 2008; van der Maaten and Hinton, 2008). Kaplan-Meier survival curves and the log-rank test were used to evaluate metastasis in patients in the high- and low-risk groups in different clinical groups. Receiver operating characteristic (ROC) curve (R package “timeROC”) and time-dependent receiver operating characteristic (T-ROC) analyses (R package “pec”) were used to evaluate the predictive ability of the model. The state of hypoxia, cellular component (CC) and molecular function (MF) in Gene Ontology (GO) and Kyoto Encyclopedia of Genes and Genomes (KEGG) pathway enrichment analyses were carried out by gene set enrichment analysis (GSEA) software (version 4.1.0) and the R package “GOplot” in different groups (Subramanian et al., 2005). Cellminer^6^ was used to explore the resistance of commonly used medicines in prostate cancer and screen sensitive small molecule medicines (Pommier et al., 2012). The degree of immune infiltration in different groups was obtained by three algorithms, namely, the ssGSEA algorithm (immune gene set downloaded from MSigDB), CIBERSORT algorithm (immune gene set download from^7^) and immunophenoscore (IPS) algorithm (Newman et al., 2015; Charoentong et al., 2017). Additionally, the immune cells were subgrouped by the previous pattern (Thorsson et al., 2018). A nomogram and calibration curve were obtained by the R package “rms,” (Zhang and Kattan, 2017) and decision curve analysis (DCA) was used to evaluate the clinical benefits to patients with the model (Vickers and Elkin, 2006).

### Cell Culture and Transfection

Prostate cancer cell lines LNCaP and DU145 were prepared from our research laboratory and cultured in DMEM and RPMI-1640 medium at 37°C with 5% CO2 (each medium containing 10% FBS). In the hypoxic group, the oxygen concentration in the cell culture environment was set to 0.5%. The hypoxic culture time was determined to be 72 h by a hypoxia exposure gradient pre-experiment.

Small interfering RNA (siRNA) against *ALDOB* (GAA GUAUACUCCAGAACAATT-UUGUUCUGGAGUAUACUUC TT) and siRNA negative control (si-NC) (UUCUCCGAACGU-GUCACGUTTACGUGACACGUUCGGAGAATT). When they reached 70–80% confluence, LNCaP and DU145 cells were transfected with 750 μL mixed solution of siRNA and Lipofectamine 3000 (Invitrogen, United States) following the instructions. Then, transfected cells were collected after 24 h of culture for the next step.

### Quantitative Real-Time Polymerase Chain Reaction (qRT-PCR)

RNA was extracted from cells by adding TRIzol reagent (Invitrogen, United States) and following the manufacturer’s instructions. Then, the purity of the RNA obtained was determined by the OD260/280 ratio, which was considered to be very pure in the range of 1.8–2.1.

PromeScript RT Master Mix (TaKaRa, Japan) was used for reverse transcription. The cDNA product was diluted 10 times to prepare a qRT-PCR system with the *ALDOB* primer and SYBR Green mix (TaKaRa, Japan). The reaction step settings were 95°C for 5 min in the holding stage, 40 cycles of 95°C for 15 s and 60°C for 20 s and 72°C for 40 s in the cycling stage, 95°C for 15 s and 60°C for 1 min and 95°C for 15 s in the melt curve stage. Using GAPDH as an internal reference, the relative expression levels of different groups were determined by the 2-ΔΔCt method (Ivak and Schmittgen, 2001).

The primers were as follows: *ALDOB* (Forward: TGGC GTGCTGTGCTGAGGAT, Reverse: CTGCTGACAGATGCTG GCGTAG), GAPDH (Forward: AGATCATCAGCAATGCCTC CT, Reverse: TGAGTCCTTCCACGATACCAA).

### Cell Proliferation Assay

LNCaP and DU145 cell lines were inoculated in a 96-well plate. Four groups were established, (a) normoxia group; (b) hypoxia group; (c) hypoxia + si-NC group; and (d) hypoxia + si-*ALDOB* group, using the culture conditions described above. After 72 h of culture, cells were incubated for 2 h with 10 μL Cell Counting Kit-8 (CCK-8) according to the reagent instructions, and 450 nm OD was detected by a microplate reader.

### Transwell Assay

After 24 h of starvation culture, four groups of cells were inoculated in the upper chamber of a 24-well Transwell chamber precoated with Matrigel (BD Bioscience, San Jose, CA, United States) for invasion assays. Then, 500 μL medium containing 20% FBS was added to the lower chamber. After 24 h of culture, the Transwell chamber was stained with crystal violet at 37°C for 30 min, and the number of invaded cells was counted at high magnification (200×).

## Results

### Research Process and Identification of Risk Factors for Hypoxia

**FIGURE 1:**
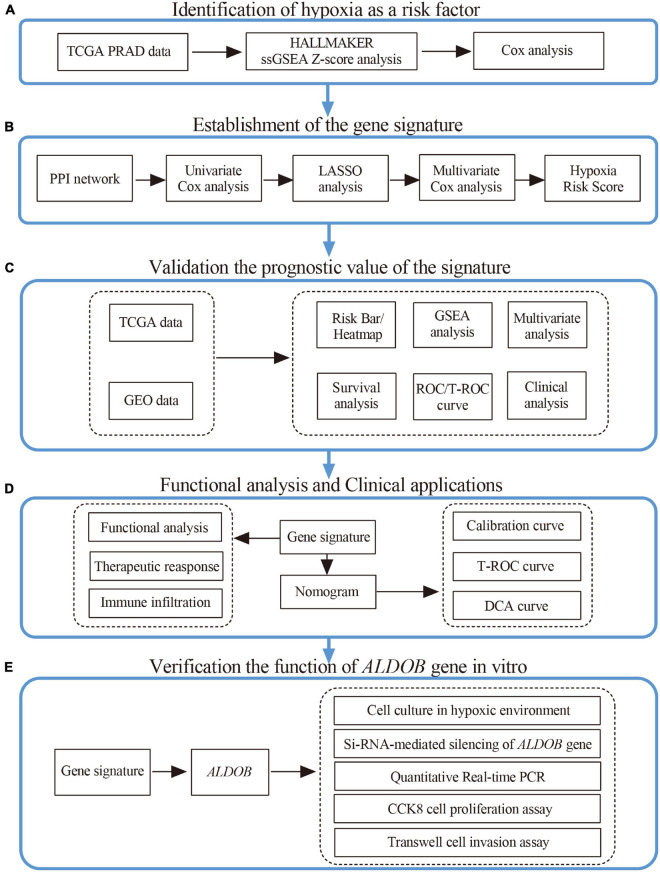
Research design and process diagram.

**FIGURE 2:**
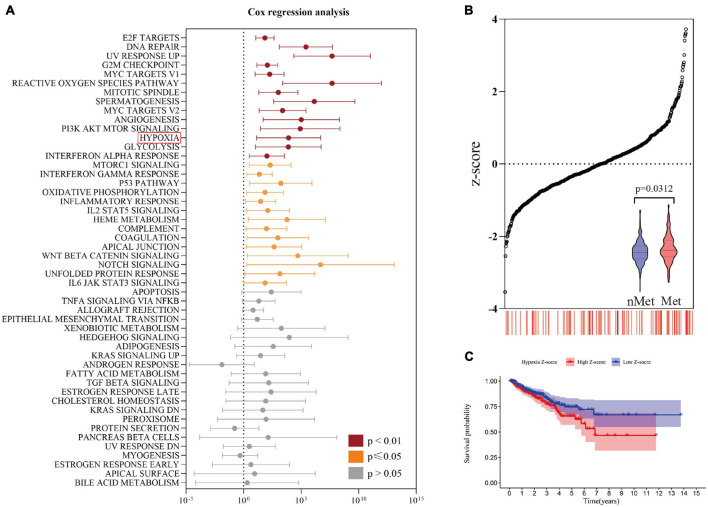
Identification of risk factors for hypoxia. **(A)** Univariate Cox regression analysis revealed a significant correlation between hypoxia and metastasis-free survival. **(B)** The number of metastatic patients increased significantly with increasing hypoxia z-score. **(C)** The prognosis of the high hypoxia z-score group was worse than that of the low hypoxia z-score group by Kaplan-Meier analysis.

The overall flow of this study is shown in Figure 1. First, the ssGSEA score among 50 pathological pathways was included in univariate Cox regression analysis to evaluate the effect of each pathological pathway on metastasis-free survival (MFS) (Supplementary File 2). As shown in Figure 2A, HYPOXIA had a great impact on MFS (*p* = 0.0059). As the hypoxia z-score increased, the number of patients with metastasis also increased (Figure 2B). Next, patients were divided into high z-score and low z-score groups according to the median z-score for hypoxia. The number of metastases in the high-risk group was higher than that in the low-risk group (*p* = 0.0312). The MFS of the high z-score group was worse than that of the low z-score group (Figure 2C). To date, hypoxia has been identified as a risk factor for prostate cancer metastasis. The clinical characteristics of the cohorts were uploaded to Supplementary File 1.

### Establishment of a Gene-Related Hypoxia Risk Score

**FIGURE 3:**
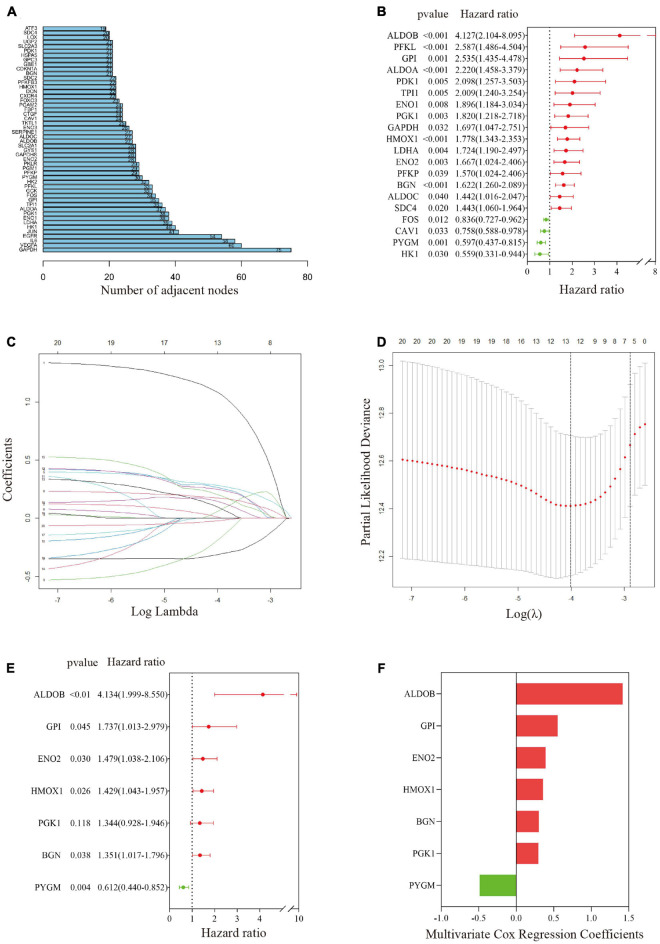
Establishment of a gene-related hypoxia risk score. **(A)** Hypoxia-related genes in the top 50 connecting nodes in the protein–protein interaction network. **(B)** Twenty hypoxia-related genes were screened by univariate Cox regression analysis. **(C,D)** After LASSO Cox regression was used to filter hypoxia-related genes, the best λ value was 0.018, and 13 indicators remained. **(E)** Final hypoxia-related gene signature obtained by multivariate Cox stepwise regression. **(F)** The coefficients of the seven genes in the signature.

A PPI network was constructed through the String PPI website, and the first 50 genes were selected according to the ranking of gene-connecting nodes (Figure 3A). Figure 3B shows 20 genes selected from the 50 genes that were significantly related to prognosis after univariate Cox regression analysis. Then, the 20 candidate genes were further screened by the LASSO Cox regression model; the λ of the best model was 0.018, containing 13 genes (Figures 3C,D). After that, the 13 genes were included in multivariate Cox stepwise regression analysis, and a predictive model composed of 7 genes was obtained (Figure 3E and Supplementary File 3); the genes were aldolase B, fructose-bisphosphate (*ALDOB*), glucose-6-phosphate isomerase (*GPI*), heme oxygenase 1 (*HMOX1*), phosphorylase glycogen muscle (*PYGM*), biglycan (*BGN*), enolase 2 (*ENO2*) and phosphoglycerate kinase 1 (*PGK1*). The coefficients of these genes are shown in Figure 3F. Except for that of the protective gene *PYGM*, the expression of the other genes was significantly correlated with predicted metastasis. Finally, the hypoxia risk score was obtained by the formula *ALDOB*^∗^ 1.419194944 + GPI^∗^ 0.552184172 + *HMOX1*^∗^ 0.35686836 -*PYGM*^∗^ 0.490233672 + *BGN*^∗^ 0.301083663 + *ENO2*^∗^ 0.39107228 + *PGK1*^∗^ 0.295317893.

### The Hypoxia Risk Score Predicted Poor Metastasis in the Training Set

**FIGURE 4:**
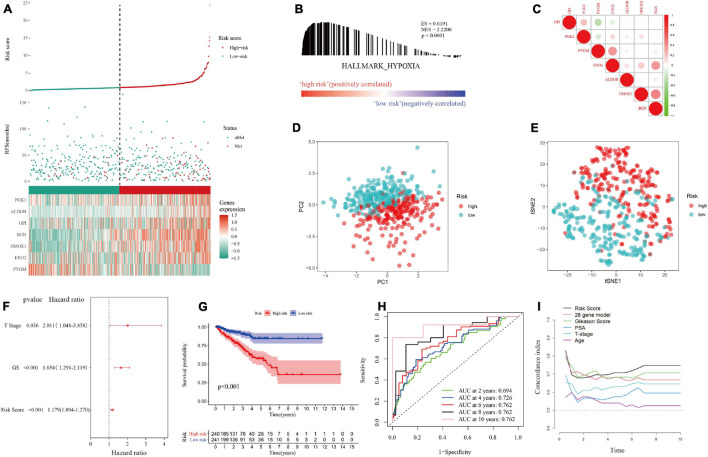
Distribution of the hypoxia risk score in the training set. **(A)** Patient survival distribution map and gene expression heat map of hypoxia-related genes at different risk scores. **(B)** GSEA proved that patients with a high risk score had a hypoxic microenvironment. **(C)** Gene correlation heat map showing low correlation among genes. **(D,E)** PCA and tSNE analysis indicated that the model could be used to distinguish between different risk groups. **(F)** The hypoxia risk score was an independent prognostic factor for metastasis-free survival. **(G)** Kaplan-Meier survival analysis confirmed that patients with a high risk of hypoxia had a worse prognosis. **(H)** The 10-year AUC determined by ROC analysis of the hypoxia gene signature was relatively high, suggesting that the predictive ability of the signature was good. **(I)** Compared with those of other models and clinical characteristics, the average AUC of the hypoxia risk score was the highest, indicating that the predictive ability of the risk score was the best.

In the training set, patients were divided into a high-risk group and a low-risk group according to the median hypoxia risk score. Obviously, the distribution of metastatic patients in the high-risk group was higher, and it was found that the genes were highly expressed only in the high-risk group, except for *PYGM*, which was highly expressed in the low-risk group (Figure 4A). GSEA and loss of *PTEN* expression (Bhandari et al., 2019) confirmed hypoxic status of tumor in the high risk group (Figure 4B; Supplementary Figure 1 and Supplementary File 4). Figure 4C reveals that there were no significant correlations among the genes in the gene signature, indicating that the model was optimized well. In addition, PCA and tSNE analysis indicated that the model could be used to distinguish between patients at different risks (Figures 4D,E). Then, combined with clinical features, the risk score was determined to be an independent prognostic factor for MFS by multivariate Cox regression analysis (Figure 4F). Kaplan-Meier survival curves showed that the MFS of patients in the high-risk group was significantly worse than that of those in the low-risk group (*p* < 0.001, Figure 4G). Figure 4H implies that the area under the curve (AUC) of the ROC curve for the hypoxia risk score within 10 years increased over time, suggesting that the average predictive ability of the hypoxia risk score was strong (AUC > 0.75). Additionally, the T-ROC curve suggested that the AUC of the risk score was higher than that of other clinical features and tended to be stable over time (Figure 4I). Furthermore, the study included a model containing 28 hypoxia-related genes for the prediction of biochemical recurrence of localized prostate cancer (Supplementary File 5) (Yang et al., 2018). The T-ROC curve also showed that over time, the curve of the hypoxia risk score was higher than that of the 28-gene model (Figure 4I).

**FIGURE 5:**
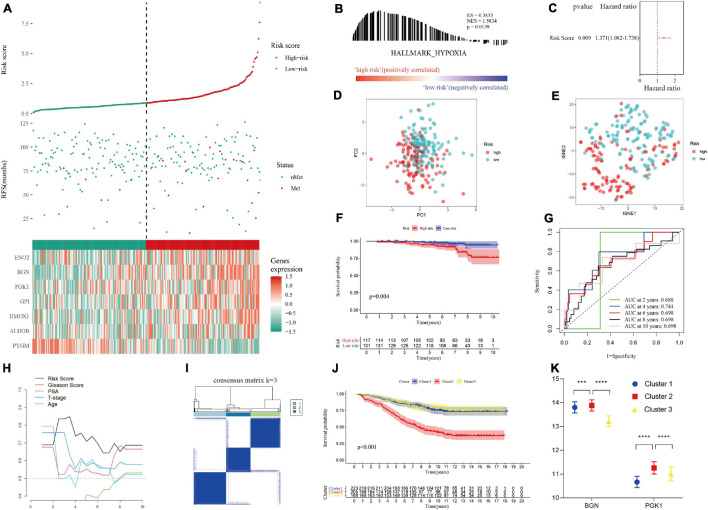
Validation of the hypoxia risk score in the test set. **(A)** Patient survival distribution map and gene expression heat map of hypoxia-related genes at different risk scores in test set I. **(B)** GSEA proved that patients in test set I with a high risk score had a hypoxic microenvironment. **(C)** The hypoxia risk score was an independent prognostic factor for metastasis-free survival in test set I. **(D,E)** PCA and tSNE analysis indicated that the model could be used to distinguish between different risk groups. **(F)** Kaplan-Meier survival analysis confirmed that patients with a high risk of hypoxia had worse metastasis-free survival in test set I. **(G)** The 10-year AUC determined by ROC analysis of the hypoxia gene signature was relatively high in test set I, suggesting that the predictive ability of the signature was good. **(H)** Compared with that of other clinical characteristics, the average AUC of the hypoxia risk score was the highest in test set I, indicating that the predictive ability of the risk score was the best. **(I)** The supplementary cohort was divided into 3 clusters with the remaining hypoxia genes according to the optimal K value. **(J)** Kaplan-Meier survival analysis showed that cluster 2 had worse metastasis-free survival than cluster 1 in the supplementary cohort. **(K)** The remaining hypoxia genes were highly expressed in cluster 2, which had the worst prognosis.

### Validation of the Hypoxia Risk Score in the Test Set

The hypoxia risk score was applied to the test set to verify the performance of the predictive model (Supplementary File 3). Encouragingly, the distribution of metastatic patients and gene expression patterns in different risk groups in test I group (GSE116918 dataset) were the same as those in the training group (Figure 5A). GSEA confirmed that patients in the high-risk group in test set I had a hypoxic microenvironment (Figure 5B and Supplementary File 4). As with the training group, PCA and tSNE analyses demonstrated good differentiability among different subgroups of patients (Figures 5D,E), and multivariate Cox regression analysis confirmed that the hypoxia risk score was an independent prognostic factor for MFS (Figure 5C). Kaplan-Meier survival analysis validated that the MFS of high-risk patients was worse than that of low-risk patients (*p* = 0.004, Figure 5F). ROC and T-ROC curves confirmed the good predictive ability of the hypoxia risk score (Figures 5G,H). However, in the supplementary GSE10645 dataset cohort, the genes in the hypoxia risk score were incomplete because of differences in the chip platforms. The cohort was divided into 3 clusters based on the remaining hypoxia genes according to the optimal *K* value by performing NMF consensus clustering (Figure 5I). Kaplan-Meier survival analysis showed that there were significant differences in the MFS among different clusters, and the MFS of cluster 2 was worse than that of cluster 1 and cluster 3 (Figure 5J). Furthermore, according to gene expression matrix analysis, there was a significant difference in the high expression of the remaining hypoxia genes in cluster 2, which had the worst prognosis (Figures 5J,K).

### A High Hypoxia Risk Score Indicated a Poor Prognosis in Different Clinical Stratifications

**FIGURE 6:**
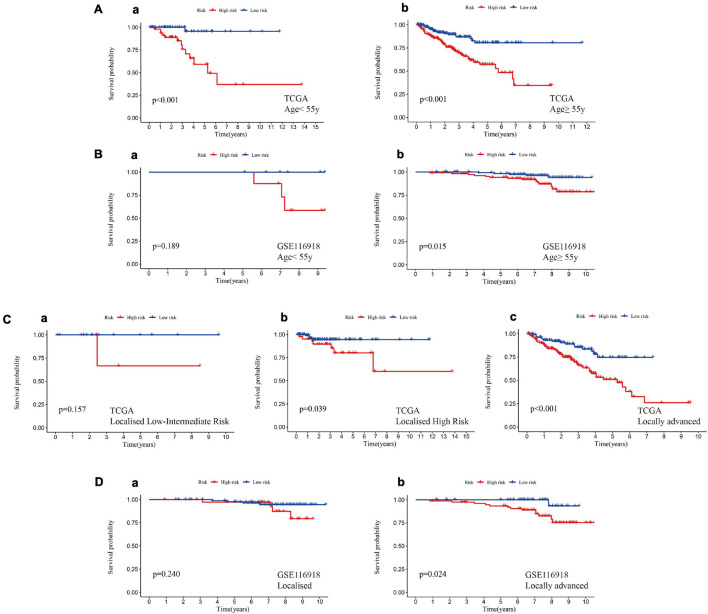
A high hypoxia risk score indicated a poor prognosis in different clinical stratifications. **(A)** A high hypoxia risk score indicated a poor MFS in early-onset prostate cancer (**a**, age < 55 years) and non-early-onset prostate cancer (**b**, age ≥ 55 years) in the training set. **(B)** A high hypoxia risk score indicated poor MFS in early-onset prostate cancer (**a**, age < 55 years) and non-early-onset prostate cancer (**b**, age ≥ 55 years) in test set I. **(C)** A high hypoxia risk score indicated a poor MFS in localized low- to intermediate-risk **(a)**, localized high-risk **(b)**, and locally advanced **(c)** prostate cancer in the training set. **(D)** A high hypoxia risk score indicated poor MFS in localized **(a)** and locally advanced **(b)** prostate cancer in test set I. Localized low-risk: PSA < 10 ng/mL, GS < 7 (ISUP grade 1) and cT1-2a. Localized intermediate risk: PSA 10–20 ng/mL, GS 7 (ISUP grade 2/3) or cT2b. Localized high-risk: PSA > 20 ng/mL, GS > 7 (ISUP grade 4/5) or cT2c. Locally advanced: any PSA, any GS (any ISUP grade), and cT3-4 or cN+.

Patients in the training set and test set I were divided into different subgroups according to different clinical characteristics (Supplementary File 6). In the age subgrouping, a high risk score indicated worse MFS in both the early-onset prostate cancer group (age ≤ 55 years) and the non-early-onset prostate cancer group (age > 55 years), which was confirmed in test set I (Figures 6A,B). However, the difference in MFS between the high-risk and low-risk groups in the early-onset prostate cancer group was not statistically significant (*p* = 0.189); this might be due to an insufficient sample size in this group. Patients were divided into stages of localized and locally advanced prostate cancer according to the European Association of Urology (EAU) risk groups. In the training set, a high risk of hypoxia predicted a worse prognosis in each stage, especially in the locally advanced stage, which had a *p* value < 0.001 (Figure 6C). In the localized low-intermediate risk stage, the difference in MFS between the high-risk group and the low-risk group was not statistically significant (*p* = 0.157), which might be caused by the reason mentioned above; that is, the sample size of this group was small. The previous results were validated in locally advanced patients in test set I, and it was noted that there was no statistically significant difference in MFS among the localized stage patients (*p* = 0.240, Figure 6D). This might be because test set I (GSE116918 dataset) includes patients who received radical radiotherapy/chemotherapy, which counteracts the hypoxic environment, thereby improving patient prognosis and prolonging MFS (Schito et al., 2020). In addition, the expression of genes in the signature was significantly positively correlated with the Gleason score and T stage (Supplementary Figure 2).

### Functional Analysis and Therapeutic Response

**FIGURE 7:**
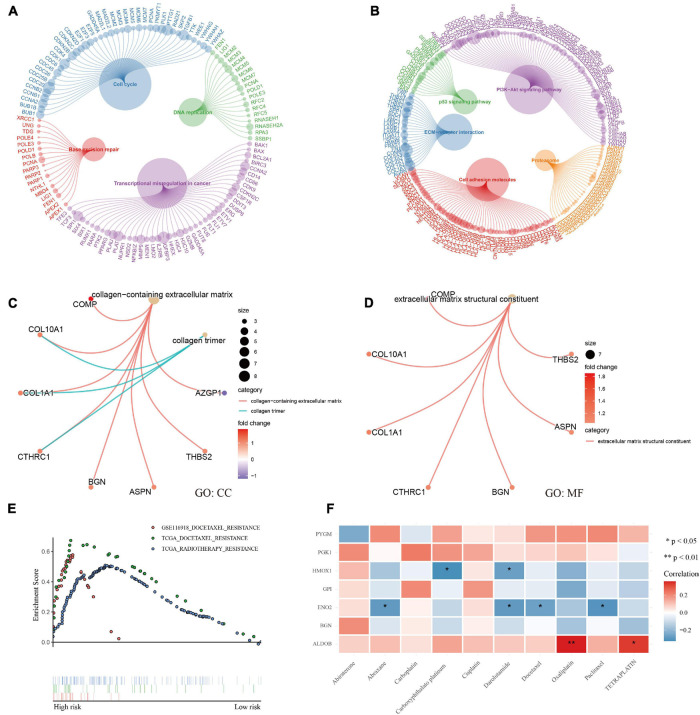
Functional analysis and therapeutic response. **(A–D)** KEGG and GO enrichment analysis showed that DNA replication, transcription, cell growth, and extracellular matrix remodeling were active and that the p53 and PI3K-Akt signaling pathways were activated in patients with a high risk of hypoxia. **(E)** GSEA showed docetaxel and radiotherapy resistance in patients with a high risk of hypoxia. **(F)** Correlation analysis between genes and common medicine sensitivity.

Enrichment analysis using the KEGG and GO databases revealed the functional differences between different groups (Supplementary File 7). Patients in the high-risk group had more exuberant DNA replication and cell growth and were more prone to base mismatch and transcriptional disorders (Figure 7A). Interestingly, in the high-risk group, in addition to the p53 and PI3K-Akt signaling pathways, the ECM-receptor interaction and cell adhesion molecule pathways were also activated (Figure 7B). The results of GO analysis also confirmed that extracellular matrix remodeling occurred in the high-risk hypoxia group (Figures 7C,D).

The study also analyzed the resistance and sensitivity to treatment. As shown in Figure 7E, patients in the high-risk group were resistant to docetaxel and radiotherapy. Based on the CellMiner database (Supplementary File 8), the correlation analysis of commonly used therapeutic medicines for prostate cancer verified the therapeutic resistance of docetaxel, and the analysis showed that the medicines were sensitive to the *ALDOB* gene, especially oxaliplatin and teraplatin (Figure 7F). Finally, the first 16 small molecular medicines that are sensitive to genes (Supplementary Figure 3) were screened, and most of the drugs showed strong sensitivity to *ALDOB*.

### Immune Cell Infiltration

**FIGURE 8:**
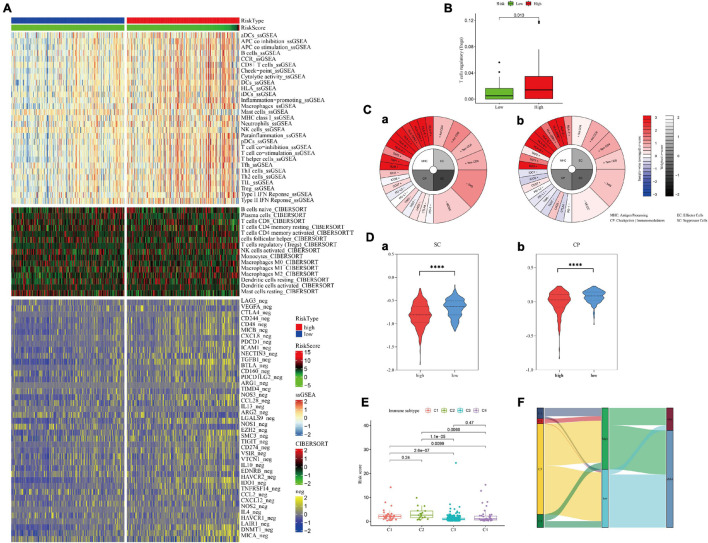
Immune cell infiltration in the high- and low-hypoxia-risk groups in the training set. **(A,B)** Negative immunoregulatory genes were highly expressed in the high-risk-score group. **(C)** IPS results of patients with the highest **(a)** and lowest **(b)** hypoxia risk scores. **(D)** The IPS algorithm showed a significant increase in SC **(a)** and CP **(b)** in the high-risk group. **(E,F)** Immunotyping results showed that C1, C2, and C4 types were increased in the high-risk group.

Considering the possible effects of hypoxia on the tumor immune environment, several different algorithms were used to explore whether there were differences in immune infiltration in different groups (Supplementary File 9). In the high-risk group, most of the negative immune regulatory genes were highly expressed (Figure 8A), and Treg cells were significantly increased (*p* = 0.013, Figure 8B). Further calculation of the immunophenotypic score confirmed the trend of immunosuppression in the high-risk group. As shown in Figures 8C,D, suppressor cells and checkpoints/immunomodulators were significantly increased in the high-risk group (*p* < 0.0001). Moreover, the results of immunophenotyping found that type C3 (good prognosis) (Thorsson et al., 2018) was clustered with a lower risk score. C1, C2, and C4 types, which represent a poor prognosis (Thorsson et al., 2018), showed an increased risk score (Figures 8E,F). Process files were uploaded to Supplementary File 9.

### Construction and Calibration of a Nomogram Including Clinical Features

**FIGURE 9:**
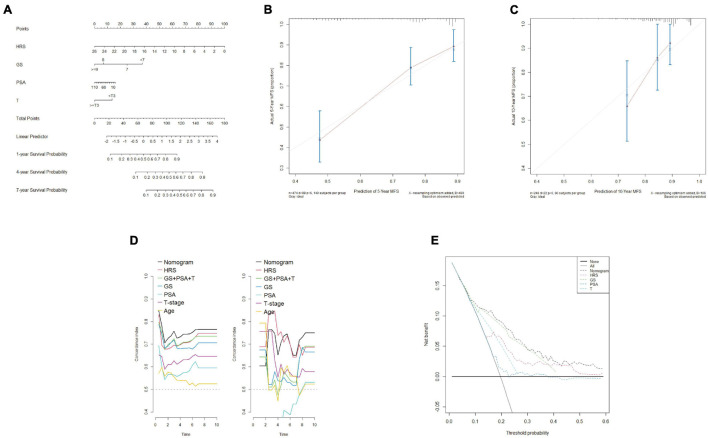
Construction and calibration of a nomogram including clinical features. **(A)** By combining the hypoxia risk score and clinical characteristics, a nomogram was constructed. **(B,C)** The 5-year calibration curve (training set) and 10-year calibration curve (test set I) showed a good degree of fit. **(D)** T-ROC analysis showed that the average AUC of the nomogram was the highest in the training set (left) and test set I (right), indicating that the predictive ability of the nomogram was the best. **(E)** DCA confirmed that the nomogram had the greatest scope of application and net benefit.

To assess the metastatic risk of prostate cancer, a nomogram was constructed using the hypoxia score and clinical features as parameters (Figure 9A). The calibration curve showed the fitting degree between the predicted probability and the actual probability of the training set at 5 years and that of the test set at 10 years, indicating that the accuracy of the nomogram was good (Figures 9B,C). T-ROC curve analysis was used to compare the predictive ability of the nomogram with that of other models and parameters. The average AUC of the nomogram was the highest, suggesting that it had the best predictive ability (Figure 9D). DCA implied that the nomogram was the most widely used and had the highest net clinical benefit compared with other clinical features (Figure 9E).

### Hypoxia-Mediated High Expression of *ALDOB* Promoted the Proliferation and Invasion of Prostate Cancer Cells *in vitro*

**FIGURE 10:**
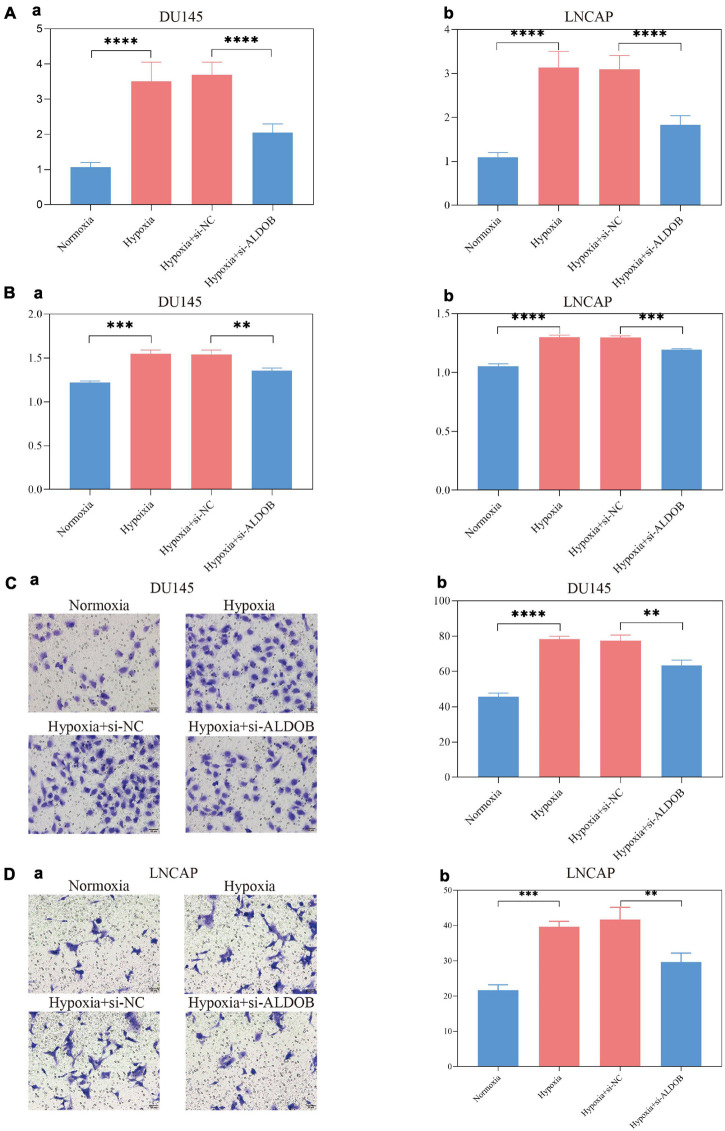
In the DU145 and LNCAP prostate cancer cell lines, the expression of ALDOB was increased under hypoxia **(A)**, promoting cell proliferation **(B)** and invasion **(C,D)**.

The *ALDOB* gene, which contributed the most to the signature and was more sensitive to most medicines, was selected for *in vitro* experiments to verify its effect on prostate cancer cells under hypoxia (Supplementary File 10). According to the sensitivity to androgen, two prostate cancer cell lines, LNCaP and DU145, were selected. After 72 h of culture, the expression of *ALDOB* in the two cell lines increased significantly under hypoxia, and after si-*ALDOB* RNA transfection, the expression was suppressed (Figure 10A). Furthermore, CCK-8 and Transwell assays confirmed that the high expression of *ALDOB* promoted the proliferation and invasion of the 2 cell lines, which were inhibited after interference (Figures 10B–D and Supplementary File 10).

## Discussion

Hypoxia is a characteristic of tumor development, often resulting from the growth rate of tumors exceeding the rate of neovascularization. The adaptation of cancer cells to an anoxic environment not only promotes the development and invasion of cancer (Han et al., 2018; Li et al., 2021) but also leads to the development of resistance to drug therapy, radiotherapy and chemotherapy and reduces the efficiency of treatment (Ranasinghe et al., 2013; Fraga et al., 2015; Casillas et al., 2018). All these effects indicate a poor prognosis. The use of oxygenation therapy can alleviate the hypoxic tumor microenvironment and achieve treatment sensitization, but only patients with a highly hypoxic microenvironment can benefit from this therapy (Krishnamachary et al., 2020). Therefore, using the expression of hypoxia-related genes to evaluate the level of tumor hypoxia can not only distinguish patients with a poor prognosis but also optimize individualized treatment.

In recent years, research on the development of hypoxia signature models has increased, and these models have been used to predict the behavior of a variety of cancers, including lung cancer, gastric cancer, hepatocellular carcinoma, melanoma, and oral squamous cell carcinoma (Suh et al., 2017; Liu et al., 2020; Mo et al., 2020; Zhang et al., 2020; Shou et al., 2021). Yang et al. (2018) developed a 28-gene hypoxia-related prognostic signature in 2018 to predict biochemical recurrence of localized prostate cancer. This is an excellent study, which is convincing and satisfactory from the design to the results. However, as the authors noted, hypoxia-related gene signatures have the potential to be further simplified. Moreover, systemic metastasis is the main cause of death in patients with prostate cancer, and the gene signature for biochemical recurrence may ignore the hypoxia-related genes that play important roles in the process of metastasis. Therefore, a new streamlined hypoxia-related gene signature needs to be developed to distinguish patients with a high risk of metastasis and to guide treatment.

Briefly, this study used advanced bioinformatics analysis algorithms to determine a hypoxia-related signature composed of seven genes, which had a strong ability to distinguish and predict patients with a high metastatic risk in different cohorts. Functional analysis revealed activation of p53, PI3K-Akt signaling pathways and active extracellular matrix remodeling at high hypoxia risk, which may be closely related to the occurrence of metastasis. Further analysis showed that there was significant treatment resistance and immunosuppression in patients with a high risk score. In addition, the most important gene in the signature, *ALDOB*, was tested *in vitro* to verify the relationship between its expression and hypoxia and its effect on prostate cancer cells. The results showed that hypoxia increased the expression of *ALDOB* and promoted the proliferation and invasion of LNCaP and DU145 cells. Finally, to quantify the risk of systemic metastasis, a nomogram combining clinical features and the hypoxia risk score was constructed. The calibration curve, T-ROC curve and DCA curve all proved the high reliability and accuracy of the nomogram.

Among the genes in the signature, *ALDOB* made the greatest contribution to the risk score. Previous studies have shown that the absence of *ALDOB* leads to the loss of Akt inhibition, promotes the development of cancer and indicates a poor prognosis (Lian et al., 2019; Sun et al., 2019; He et al., 2020). This seems to be contrary to the conclusions of this study, but there are also studies that support the conclusions of this research. The expression of *ALDOB* is upregulated in liver metastatic tumor cells, and the upregulation of glucose metabolism provides energy for metastatic tumor cells, resists apoptosis and autophagy, inhibits oxidative stress, and maintains tumor cell proliferation under severe hypoxia, while a low-fructose diet significantly reduces the growth of liver metastatic cells (Chae et al., 2016; Bu et al., 2018; Leong, 2018). Are these lines of evidence contradictory? Further analysis in this study led to a reasonable inference. By comparing the expression of *ALDOB* between normal prostate cells and prostate cancer cells, it was found that *ALDOB* was highly expressed in normal tissues, while *ALDOB* expression was very low in tumor tissues (Supplementary Figure 4). However, as the Gleason score and T stage increased, the expression of *ALDOB* was upregulated at a very low level, which might be particularly obvious in liver metastatic tumors, but it was still far below the level of expression in normal tissues. The above results suggested that low expression of the *ALDOB* gene might be a characteristic of cancer tissue, and upregulation on the basis of this low expression level might indicate cancer metastasis, especially liver metastasis. In addition, based on the results of medicine sensitivity analysis, *ALDOB* has great potential as a targeted marker.

This study suggests that extracellular matrix remodeling is an important pathway for distant metastasis. *BGN* is a proteoglycan in the extracellular matrix that undergoes hormone-dependent regulation, and its expression is closely related to the level of androgen receptor (Jacobsen et al., 2017). In addition, 17-estradiol (E2) signaling positively regulates *BGN* expression (Majumdar et al., 2019). This may provide a continuing impetus for the invasion and metastasis of prostate cancer during the androgen deprivation stage, and high expression of *BGN* may indicate that prostate cancer has evolved into CRPC or metastasized. *HMOX1* encodes heme oxygenase-1 (HO-1), which can maintain the stability of prostate cancer cells. *HMOX1* has a protective effect on androgen-dependent prostate cancer cells during androgen deprivation therapy, promoting the transformation into androgen-independent prostate cancer cells and overexpression (Zhang et al., 2021). It is worth noting that key bone markers were significantly upregulated in prostate cancer cells cocultured with primary mouse osteoblasts induced by HO-1, which proved that *HMOX1* plays an important role in bone metastasis of prostate cancer.

A hypoxic environment can inhibit immunity and promote the invasion and metastasis of cancer (Mo et al., 2021). The conclusion of this study is consistent with these effects: in the high hypoxia score group, the negative immunoregulatory genes were in a state of high expression, while a large number of Treg cells had infiltrated. Notably, *PYGM*, which is underexpressed in invasive cancer (Dieci et al., 2016), was found to be involved in the activation and proliferation of T cells (Llavero et al., 2015). This process weakens the immune surveillance of invasive cancer cells in hypoxic environments. Experiments have shown that hypoxia-targeted therapy can restore T cell infiltration and make prostate cancer sensitive to immunotherapy (Jayaprakash et al., 2018). Therefore, *PYGM*, which is not only related to hypoxia but also involved in T cell activation, is expected to become an effective therapeutic target.

There were some limitations to this study. First, different patients may have been treated differently, which could affect gene expression, in turn could biasing the data analyzed to construct the signature, or affecting the comparability of end points across all cohorts. To reduce this potential bias, more rigorous prospective cohort studies need to be designed. Second, this study only conducted *in vitro* phenotypic experiments for *ALDOB*, and further studies on other genes and mechanisms need to be carried out. Most importantly, there is no doubt that any newly established nomogram, regardless of its reliability and predictive power, should be verified in large-scale basic trials and prospective clinical studies before clinical application.

Limitations cannot belittle virtues, and this study is the first to use hypoxia gene characteristics to predict metastasis of prostate cancer. In addition, the study explained the therapeutic response and immune infiltration observed under hypoxia and speculated on the possible mechanism of hypoxia gene characteristics.

## Conclusion

Briefly, this study screened the characteristics of hypoxia genes to predict prostate metastasis, and with further inclusion of prostate-specific clinical features, a nomogram was established to quantify the risk of metastasis. This work can not only help to identify patients with a high risk of metastasis to begin individualized treatment as early as possible but also provide a new possible therapeutic target for the prevention and treatment of prostate cancer metastasis and new ideas for future research.

## Data Availability Statement

Publicly available datasets were analyzed in this study. This data can be found here: The Cancer Genome Atlas (TCGA), https://portal.gdc.cancer.gov/; Gene Expression Omnibus database (GEO), GSE116918, https://www.ncbi.nlm.nih.gov/geo/query/acc.cgi?acc=GSE116918; Gene Expression Omnibus database (GEO), GSE10645, https://www.ncbi.nlm.nih.gov/geo/query/ac c.cgi.

## Ethics Statement

Ethical review and approval was not required for the study on human participants in accordance with the local legislation and institutional requirements. Written informed consent for participation was not required for this study in accordance with the national legislation and the institutional requirements.

## Author Contributions

HX conducted data search, download, collation, statistics and analysis, designed and carried out *in vitro* experiments, as well as manuscript writing and revision. XG, ZL, and JL helped to search the data. QY gave guidance and help in the experiment. JW and ML gave financial support to the research. ML and JW reviewed the manuscript.

## Conflict of Interest

The authors declare that the research was conducted in the absence of any commercial or financial relationships that could be construed as a potential conflict of interest.

## Publisher’s Note

All claims expressed in this article are solely those of the authors and do not necessarily represent those of their affiliated organizations, or those of the publisher, the editors and the reviewers. Any product that may be evaluated in this article, or claim that may be made by its manufacturer, is not guaranteed or endorsed by the publisher.

## Footnotes

1
https://www.ncbi.nlm.nih.gov/geo/query/acc.cgi?acc=GSE116918

2
https://www.ncbi.nlm.nih.gov/geo/query/acc.cgi?acc=GSE10645

3
http://www.gsea-msigdb.org/gsea/msigdb/search.jsp

4
https://www.string-db.org/

5
http://www.r-project.org

6
https://discover.nci.nih.gov/cellminer/-home.do

7
https://cibersortx.stanford.edu/

## Supplementary Material

Supplementary Figure 1 *PTEN* expression decreased significantly in the high risk group of training cohort **(A)** and test I cohort **(B)**.

Supplementary Figure 2 Correlations between hypoxia-related genes and clinical characteristics. **(A,B)** In the training set (left) and test set I (right), the expression of genes increased with increasing Gleason score and T stage, while the expression of PYGM showed the opposite trend.

Supplementary Figure 3 The first 16 medicines significantly related to the genes were screened by medicine sensitivity analysis based on the CellMiner database.

Supplementary Figure 4 Expression of *ALDOB* in normal and cancerous prostate tissues.
