# Supplementary figures and images for: Identification of a Hypoxia-Related Gene Signature for Predicting Systemic Metastasis in Prostate Cancer
Source: Front Cell Dev Biol. 2021 Oct 13;9:696364. doi: 10.3389/fcell.2021.696364 (PMC8548828; doi:10.3389/fcell.2021.696364)

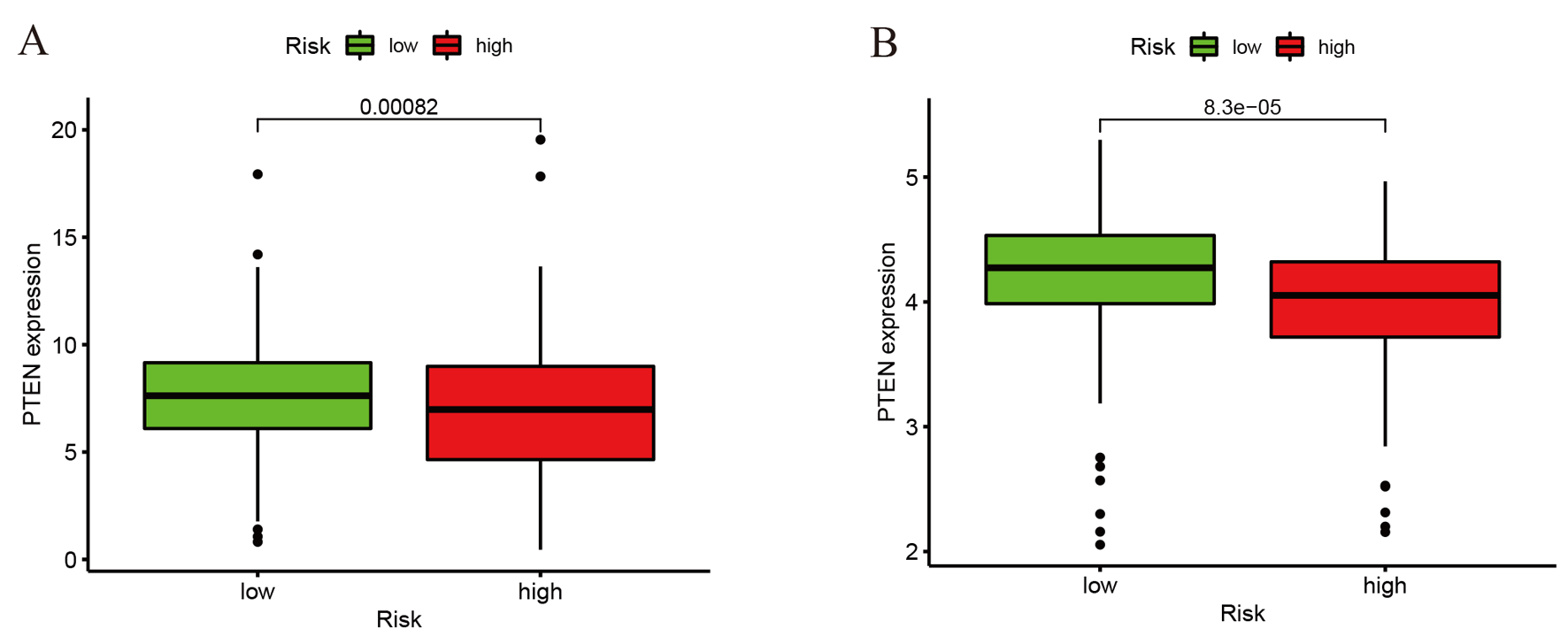

Supplement: Supplementary Figure 1 — PTEN expression decreased significantly in the high risk group of training cohort (A) and test I cohort (B). [file Image_1.TIF]

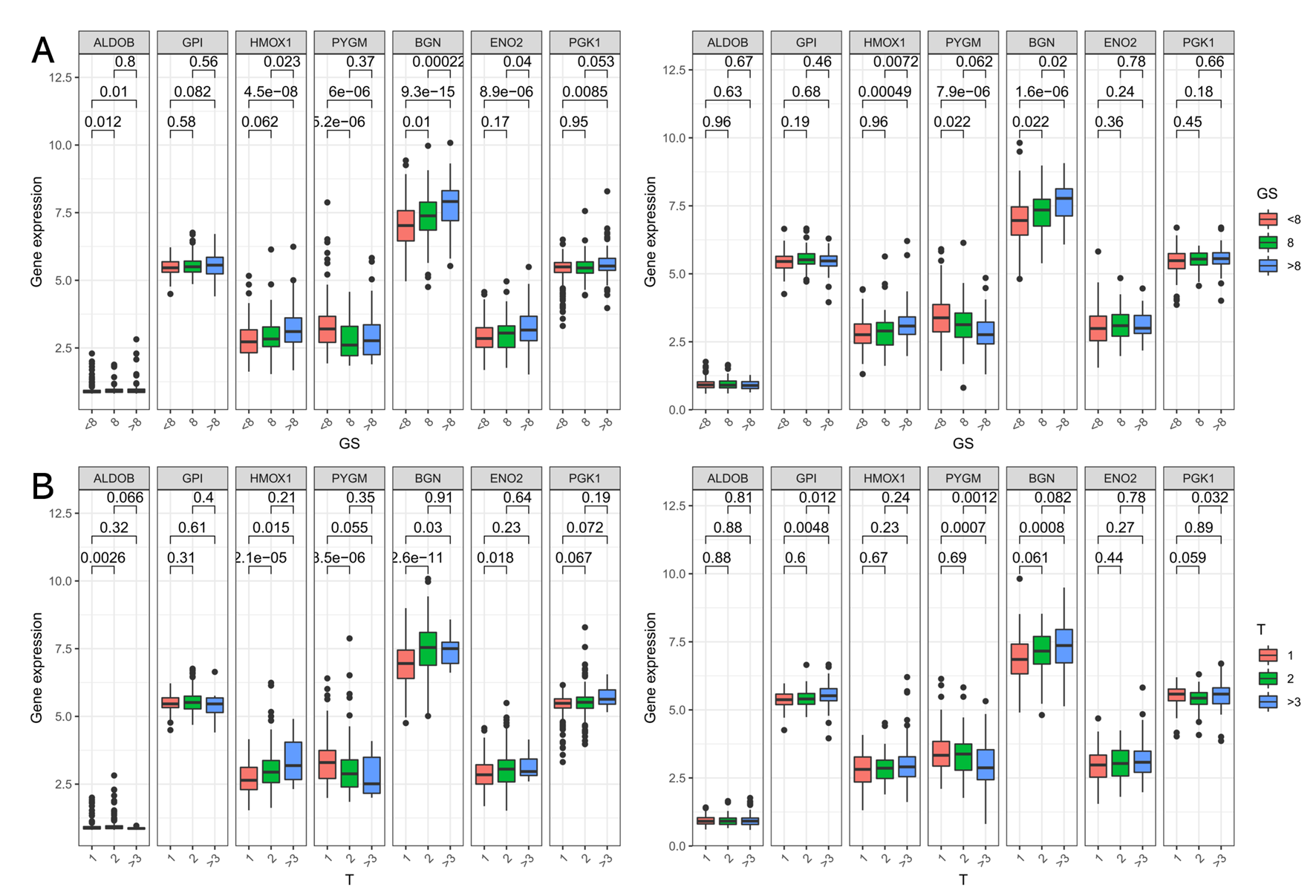

Supplement: Supplementary Figure 2 — Correlations between hypoxia-related genes and clinical characteristics. (A,B) In the training set (left) and test set I (right), the expression of genes increased with increasing Gleason score and T stage, while the expression of PYGM showed the opposite trend. [file Image_2.TIF]

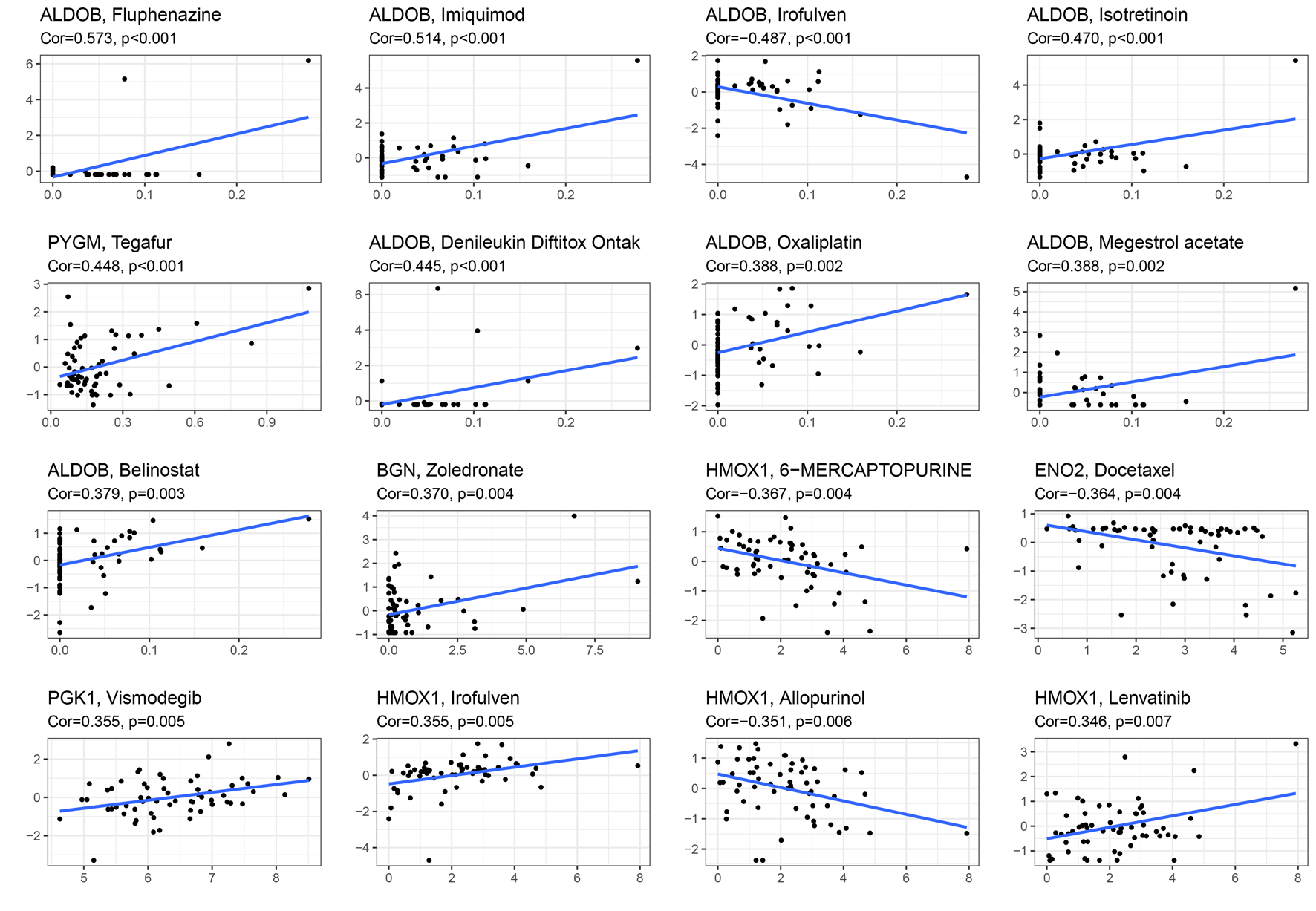

Supplement: Supplementary Figure 3 — The first 16 medicines significantly related to the genes were screened by medicine sensitivity analysis based on the CellMiner database. [file Image_3.TIF]

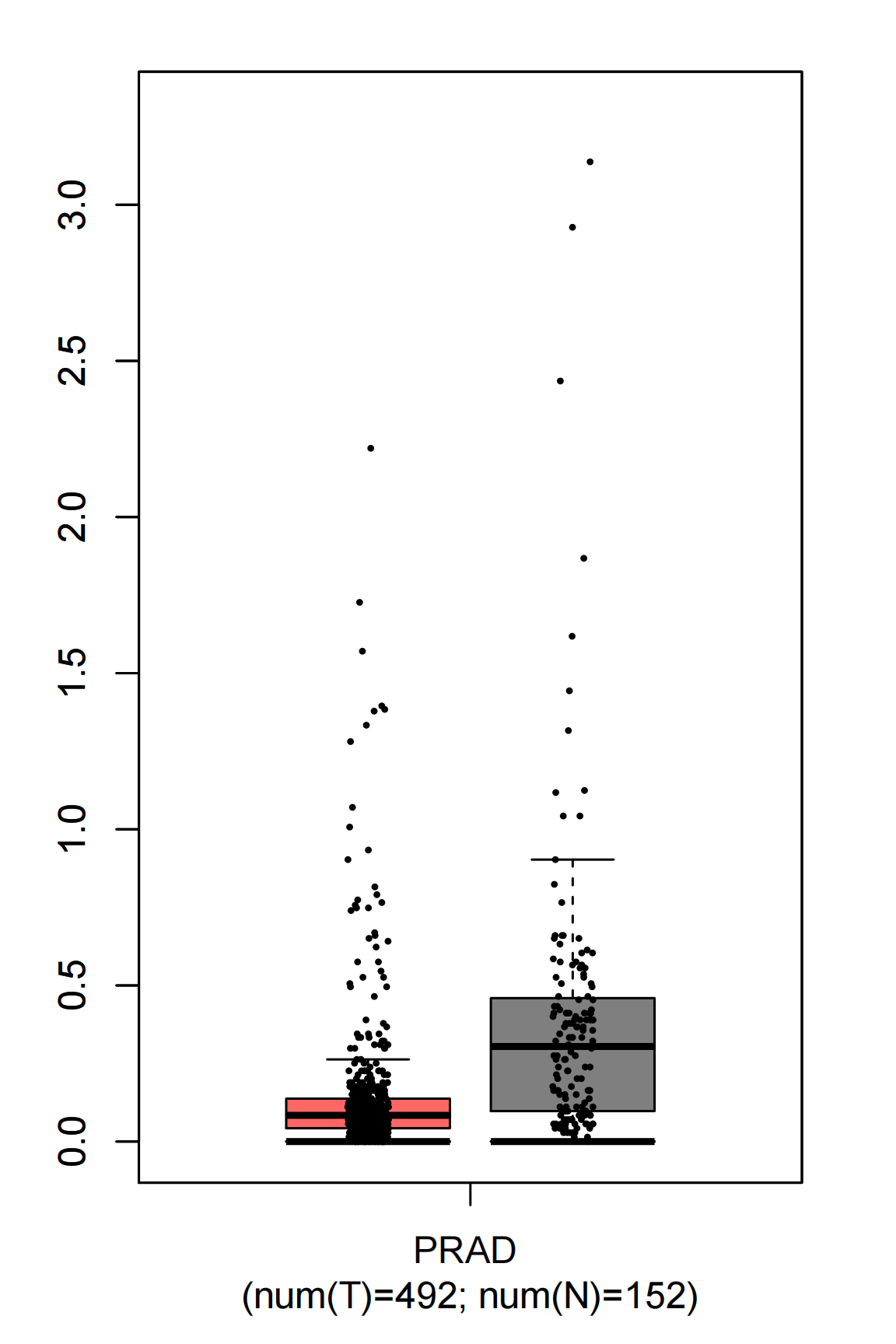

Supplement: Supplementary Figure 4 — Expression of ALDOB in normal and cancerous prostate tissues. [file Image_4.TIF]
